# Primary Mucinous Carcinoma of the Skin

**Published:** 2013-06-18

**Authors:** Jennifer Maerki, Shahida Ahmed, Edward Lee

**Affiliations:** ^a^Department of Pathology and Laboratory Medicine; ^b^Department of Surgery, University of Medicine and Dentistry of New Jersey, Newark; ^c^Department of Pathology and Laboratory Medicine; ^d^Department of Surgery, Veterans Affairs New Jersey Healthcare System, East Orange

## DESCRIPTION

The patient is an 89-year-old man presenting with a 5-year history of a slow growing chin lesion. The lesion appeared lobulated with overlying telangiectasia and measured 2.5 cm × 1.9 cm × 1.8 cm. On palpation, the lesion was firm and mobile. There was no oral mucosal involvement. On computed tomographic scan, the mass appeared well circumscribed and partially cystic, extending to the cortical margin of the mandible without evidence for periosteal reaction or cortical destruction.

Microscopically, the tumor shows lakes of mucin with small basophilic tumor cell clusters, consistent with primary mucinous carcinoma of the skin (PMCS). Rosai^1^ suggests that the ducts proliferate until the overproduction of mucin creates islands of tumor cells, essentially floating in mucinous pools. Immunostaining is positive for mucin, cytokeratin 7 (CK7), and negative for cytokeratin 20 (CK20), caudal-related homeobox gene 2 (CDX-2), and thyroid transcription factor-1 (TTF-1), ruling out gastrointestinal and lung origin and confirming a primary cutaneous origin. The tumor is incompletely excised, and the patient will follow up for an excision with additional margins.

## QUESTIONS

1. **Where does this rare cutaneous tumor commonly arise?**
2. **What is the prognosis and reoccurrence rate?**
3. **What should be included within the differential diagnosis, and how can we differentiate between these histologically?**
4. **What is the current recommended treatment?**

## DISCUSSION

Primary mucinous carcinoma of the skin is an extremely rare cutaneous cancer derived from the sweat glands. Fewer than 150 cases have been reported in the literature published in English.^2^^-^16 The tumor was first described by Lennox et al^17^ in 1952 and later designated by Mendoza and Helwig^18^ in 1971. Primary mucinous carcinoma of the skin is slightly more common in men and occurs most frequently between the ages of 50 and 70 years. Anatomically, the eyelid is most commonly affected (41%).^19^ Additional locations include the scalp (17%), face (14%), axilla (9%), chest/abdomen (7%), vulva (4%), neck (2%), extremity (2%), canthus (2%) groin (1%), and ear (1%).^19^ Our case falls into the few categorized as a primary facial lesion. Clinically, a PMCS has been described as a slow-growing, painless, nodular, red/gray/purple lesion that may be ulcerated, crusted, or with telangectasis.^15^

Mucinous carcinoma of the skin has a relatively good prognosis with rare distant metastases, but there is a high local recurrence rate of 29.4% of cases.^15^ The low metastatic potential has been attributed to the avascular characteristic of the tumor.^15^

The precise number of PMCS may be higher than previously mentioned because of the lesion frequently being mistaken for a benign tumor. When attempting to diagnose PMCS, the differential must include metastatic mucinous carcinoma, particularly of the breast and gastrointestinal tract, and mucinous basal cell carcinoma. In addition to immunostaining (CK7, CK20, P63, CK5/6, CDX-2, TTF-1, and mucin) for confirmation of tumor origin, histologic features may aid in differentiating primary versus metastatic tumors. Primary lesions tend to have more organized epithelial cells, few mitoses, and less hyperchromasia, and metastatic cells are more likely to be seen at the nodule margins. In addition, dirty necrosis is frequently found in intestinal mucinous carcinomas involving the skin.

Mucinous carcinoma is found to be resistant to chemotherapy and radiation.^19^ Therefore, the current treatment remains excision with at least 1-cm margins since there is a high local recurrence rate.^18^

## Figures and Tables

**Figure 1:**
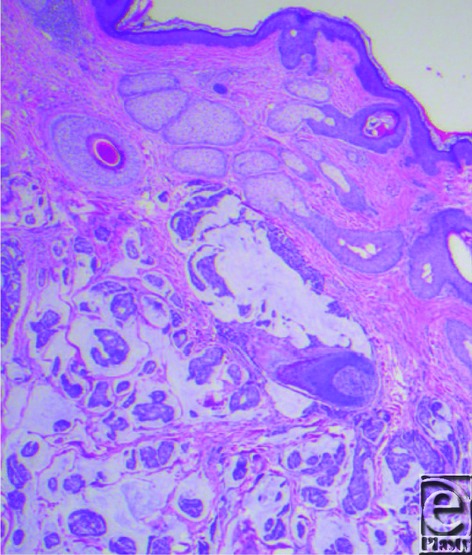
Hemotoxylin and eosin (50×).

**Figure 2:**
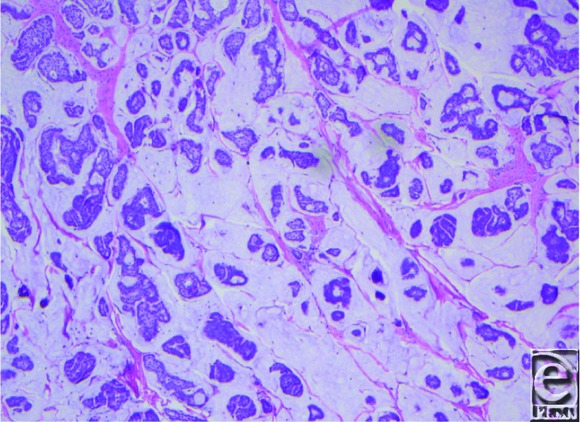
Hemotoxylin and eosin (100×).

**Figure 3:**
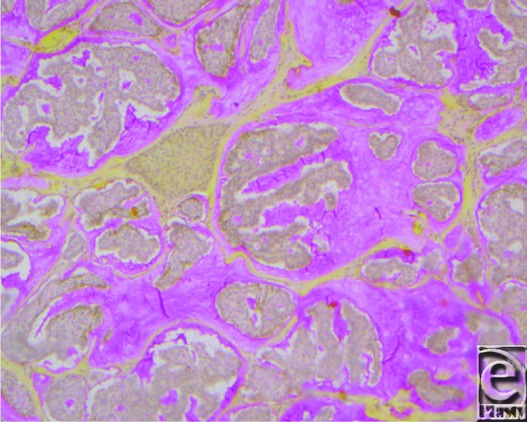
Positive mucin stain.

**Figure 4:**
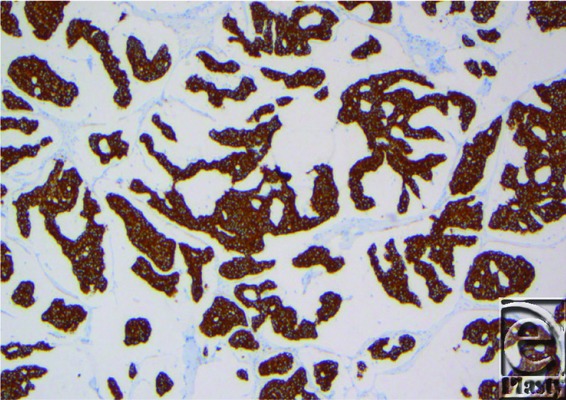
Tumor cells are positive for CK7.
